# Supplementary material for: Validating Methylated HOXA9 in Bronchial Lavage as a Diagnostic Tool in Patients Suspected of Lung Cancer
Source: Cancers (Basel). 2021 Aug 22;13(16):4223. doi: 10.3390/cancers13164223 (PMC8393370; doi:10.3390/cancers13164223)
Supplement: Supplementary file 1 [file cancers-13-04223-s001.zip › cancers-1306831-supplementary.pdf]

cancers MDPI

Supplementary Materials: Validating Methylated HOXA9 in Bronchial Lavage as a Diagnostic Tool in Patients Suspected of Lung Cancer

Sara W. C. Wen, Rikke F. Andersen, Kristian Rasmussen, Caroline Brenner Thomsen, Torben Frøstrup Hansen, Line Nederby, Henrik Hager, Anders Jakobsen and Ole Hilberg

S1. Definition of Patient Characteristics

Employment status was evaluated from the medical record. If the participant was older than 65, he/she was considered to be retired, unless otherwise specified in the medical record. Smoking status was categorized as never if the participant had smoked less than one pack year in his/her lifetime, and as ever if he/she had smoked more than one pack year. One pack year was defined as 20 cigarettes per day for a year or the equivalent in other tobacco types. Performance status was categorized as defined by the Eastern Cooperative Oncology Group. FEV1 was the forced expiratory volume of air in 1 s recorded in liters. Any comorbidity was defined as the participant having any condition that required regular medication. Cancer within five years was defined as any diagnosis of malignancy within the preceding five years, excepting non-melanoma skin cancer and carcinoma in situ cervix uteri, while previous lung cancer was defined as any previous diagnosis of lung cancer. All clinical participant characteristics were recorded at the first doctor's appointment.

S2. Primer and Probe Sequences for Droplet Digital PCR Analysis of Methylated HOXA9

Methylated HOXA9 was analyzed with an in-house ddPCR assay. (Primers and probe were obtained from LGC Biosearch Technologies, Aarhus, Denmark).

Primer sequence:

Forward	GAGTATTTCGATTTTAGTTCGTGT
Reverse	CGCGTACACTAAATTCCAC

Probe sequence:

Probe	FAM-TTAGTTTAAGGCGACGGTGTT-BHQ-1

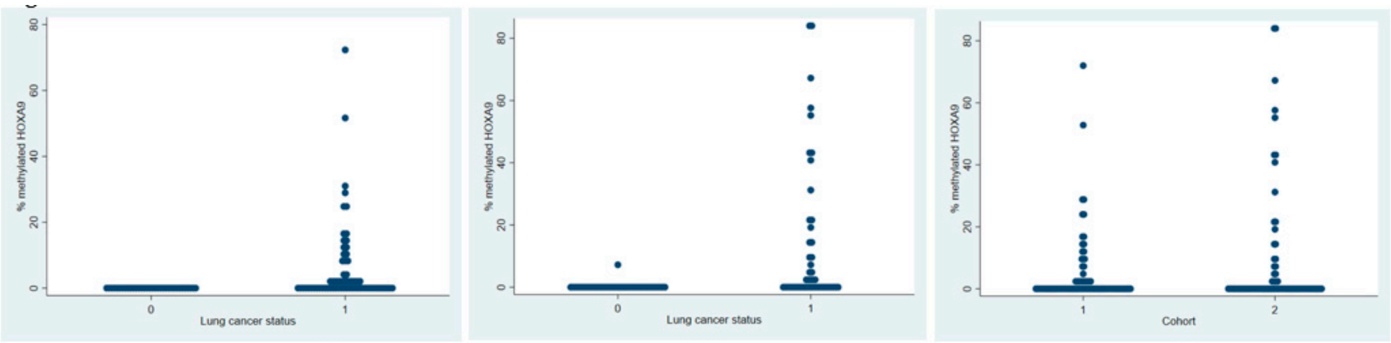

(a) (b) (c)

Figure S1. Methylated *HOXA9* in bronchial lavage fluid from participants examined for lung cancer. Dot plots visualizing the levels of methylated HOXA9 in (**a**) the Discovery cohort, (**b**) the Validation cohort and (**c**) both cohorts. In panels (**a**) and (**b**), 0 represents the controls while 1 represents the cases. In panel (**c**), the Discovery and Validation cohorts are represented by 1 and 2, respectively.

S3. Excluded Participants

Table S1. Participants excluded because they had cancers other than lung cancer. Participants diagnosed with cancer types other than lung cancer were excluded from the statistical analyses. The cancer type and methylated HOXA9 status are reported for the three patients in the Discovery cohort and the five patients in the Validation cohort.

Discovery Cohort		Validation Cohort	
**Cancer Type**	**HOXA9 Status**	**Cancer Type**	**HOXA9 Status**
Colon cancer	Positive	Mesothelioma (1)	Negative
Breast cancer	Positive	Mesothelioma (2)	Negative
T cell leukemia	Negative	B cell lymphoma	Positive
-	-	Renal cancer	Negative
-	-	Breast cancer	Positive

S4. Alternative Discovery Cohort

The Discovery cohort included participants with a previous diagnosis of lung cancer, while these participants were excluded from the Validation cohort. We provide Table S2 in order to enable the reader to compare the Discovery cohort with and without the participants with a previous lung cancer.

Table S2. Diagnostic measures. Methylated HOXA9 as a diagnostic biomarker for lung cancer reported for the Discovery and Validation cohorts, respectively. Discovery cohort A includes all eligible participants as presented in the results. Discovery cohort B excluded all participants with a previous diagnosis of lung cancer. HOXA9+ indicates detectable methylated HOXA9 (≥ 0.13%) in the bronchial lavage sample and hence a positive test. HOXA9− indicates a negative test with no detectable methylated HOXA9 (< 0.13%). Sensitivity, specificity, positive predictive value (PPV) and negative predictive value (NPV) are reported with 95% confidence intervals (CI).

Title	Discovery Cohort A			Discovery Cohort B			Validation Cohort		
**Diagnosis**	**HOXA9+**	**HOXA9−**	**Total**	**HOXA9+**	**HOXA9−**	**Total**	**HOXA9+**	**HOXA9−**	**Total**
Lung cancer	49	18	67	36	7	43	40	10	50
No lung cancer	5	29	34	4	24	28	11	34	45
Total	54	47	101	40	31	71	51	44	95
Sensitivity (95% CI)	73.1% (60.9%–83.2%)			83.7% (69.3%–93.2%)			80.0% (66.3%–90.0%)		
Specificity (95% CI)	85.3% (68.9%–95.0%)			85.7.6% (67.3%–96.0%)			75.6% (60.5%–87.1%)		
PPV (95% CI)	90.7% (79.7%–96.9%)			90.0% (76.3%–97.2%)			78.4% (64.7%–88.7%)		
NPV (95% CI)	61.7% (46.4%–75.5%)			77.4% (58.9%–90.4%)			77.3% (62.2%–88.5%)		

S5. Simple Logistic Regression Analysis

Simple logistic regression analysis was performed on all clinical characteristics and on methylated HOXA9 status in order to assess their predictive impact. The odds ratio (OR), 95% confidence interval (CI) and the test significance are reported below.

Table S3. Univariate logistic regression analyses. Simple logistic regression analyses for the Discovery and Validation cohorts, respectively. Odds ratios (OR) and p-values are presented. N/A: Previous lung cancer was introduced as an exclusion criterion in the Validation cohort. * Statistically significant impact. HOXA9 status B = the Discovery cohort without the participants with a previous diagnosis of lung cancer.

Title	Discovery Cohort			Validation Cohort		
**Variable**	**OR**	**95% CI**	***p*-Value**	**OR**	**95% CI**	***p*-Value**
Age, years	1.06	1.02-1.11	0.002 *	1.08	1.03-1.13	0.001 *
Sex, male	1.10	0.48-2.53	0.823	0.67	0.30-1.52	0.341
Smoking status	-	-	-	-	-	-
Never (reference)	1	-	-	1	-	-
Ever	1.84	0.56-5.97	0.312	9.75	2.06-46.14	0.004 *
Pack years	1.02	0.99-1.04	0.061	1.02	1.00-1.04	0.017 *

Performance status	-	-	-	-	-	-
0 (reference)	1	-	-	1	-	-
1	2.11	0.81-5.49	0.125	0.88	0.32-2.41	0.807
2	1.69	0.51-5.59	0.391	2.21	0.40-12.22	0.365
FEV1, liter	0.46	0.27-0.78	0.004 *	0.52	0.30-0.91	0.021 *
Cancer within 5 years	1.76	0.71-4.36	0.221	1.56	0.35-6.91	0.562
Previous lung cancer	2.60	0.95-7.18	0.064	N/A	N/A	N/A
Diameter, mm	1.04	0.99-1.10	0.093	1.02	1.00-1.05	0.028*
Localization	-	-	-	-	-	-
Central (reference)	1	-	-	1	-	-
Intermediate	1.18	0.17–8.02	0.862	1.00	0.30-3.38	1.000
Peripheral	0.68	0.15–3.09	0.622	0.73	0.21–2.53	0.616
HOXA9 status	15.79	5.30–47.06	<0.001 *	12.36	4.68-32.64	<0.001 *
HOXA9 status B	30.86	8.14–116.98	<0.001 *	-	-	-

S6. Clinical Multiple Logistic Regression Analysis

The model was developed based on data from the Discovery cohort. As described in the manuscript, the number of predictors were limited to a total of three due to the small sample size. We chose to include age and smoking status, as they are often among the selection criteria for lung cancer screening.

Table S5. Clinical multiple logistic regression model for prediction of lung cancer. Multiple logistic regression model developed on data from the Discovery cohort and subsequently applied to data from the Validation cohort. The model included data on 101 and 95 participants from the Discovery and Validation cohorts, respectively.

**Title**	**Discovery Cohort**			**Validation Cohort**		
**Variables**	**Odds Ratio**	**95% CI**	***p*-Value**	**Odds Ratio**	**95% CI**	***p*-Value**
Age, years	1.063	1.021–1.106	0.003	1.065	1.014–1.119	0.012
Smoking status	-	-	-	-	-	-
Never	1	-	-	1	-	-
Ever	1.417	0.404–4.976	0.586	5.895	1.167–29.786	0.032
Constant	0.025	0.002–0.383	0.008	0.004	<0.001–0.115	0.001

S7. Biomarker Multiple Logistic Regression Analysis

The model was developed on data from the Discovery cohort. Methylated HOXA9 status was included, along with age and smoking status from the clinical model.

Table S4. Biomarker multiple logistic regression model for prediction of lung cancer. Multiple logistic regression model developed on data from the Discovery cohort and subsequently applied to data from the Validation cohort. The model included data on 101, 71, and 95 participants from the Discovery cohorts A and B and the Validation cohort, respectively.

**Title**	**Discovery Cohort A**			**Discovery Cohort B**			**Validation Cohort**		
**Variables**	**OR**	**95% CI**	***p*-Value**	**OR**	**95% CI**	***p*-Value**	**OR**	**95% CI**	***p*-Value**
Age, years	1.058	1.008–1.111	0.023	1.083	1.010–1.161	0.024	1.071	1.011–1.136	0.020
Smoking status									
Never	1			1			1		
Ever	1.071	0.242–4.739	0.928	0.868	0.145–5.207	0.877	5.198	0.820–32.946	0.080
HOXA9 status	14.266	4.619–44.062	<0.001	29.869	6.898–129.329	<0.001	11.945	4.106–34.746	<0.001
Constant	0.013	<0.001–0.412	0.014	0.002	<0.001–0.231	0.011	0.001	<0.001–0.052	0.001
